# The Impact of Endograft Selection on Mid-Term Outcomes in Female Patients Following Endovascular Aortic Aneurysm Repair (EVAR) for Abdominal Aortic Aneurysm (AAA)

**DOI:** 10.7759/cureus.14584

**Published:** 2021-04-20

**Authors:** Ian P Barry, Luke P Turley, Angel R Thomas, Mariah T Mwipatayi, Bibombe P Mwipatayi

**Affiliations:** 1 Vascular Surgery, Royal Perth Hospital, Perth, AUS; 2 Surgery, Royal College of Surgeons in Ireland, Dublin, IRL; 3 Curtin Medical School, Faculty of Health Sciences, Curtin University, Perth, AUS; 4 Medicine, University of Buckingham Medical School, Buckingham, GBR; 5 Surgery, Faculty of Medicine, Dentistry and Health Sciences, University of Western Australia, Perth, AUS

**Keywords:** abdominal aortic aneurysms, endovascular stent graft, endovascular aneurysm repair, management of abdominal aortic aneurysms, aortic aneurysm repair, cardiothoracic & vascular surgery research, female gender

## Abstract

Background

Abdominal aortic aneurysms (AAA) are far more common in male than female gender, although they appear to have a more aggressive pathophysiology in females. Given the lower incidence of AAA in females, it has been difficult to assess the impact of graft selection for endovascular aortic aneurysm repair (EVAR) in this cohort.

Purpose

To identify whether graft selection influences outcomes following AAA endoluminal repair in female patients.

Methodology

A retrospective analysis of published data for 711 female patients was conducted, collating data from three cohorts - Endurant Stent Graft Natural Selection Global post-market registry (ENGAGE), Global Registry for Endovascular Aortic Treatment (GREAT) and U.S. Zenith multicenter trial in combination with the Zenith female registry. Patients were recruited into the ENGAGE registry between 2009 and 2011, the GREAT registry between August 2010 and October 2016, and into the Zenith registry between 2000 and 2003. Patients from ENGAGE received the Medtronic Endurant stent graft for infrarenal AAA repair, patients analysed in GREAT received the Gore Excluder stent graft and the Zenith group received the Cook Zenith stent graft. Analyses were performed to evaluate all-cause mortality, aorta-related mortality, endoleak occurrence and surgical reintervention rates between the three cohorts.

Results

Of the 711 females, 133 were from ENGAGE (mean age 76 years), 538 were from GREAT (mean age 75 years) and 40 were from Zenith (mean age 74 years). The rates of co-morbidities between the three groups were broadly similar except for atherosclerotic disease which was more commonly observed in those treated with the GORE Excluder. The rate of endoleaks was lower when the Excluder stent was utilised as compared to the other two stents (Excluder 6.7% vs. Zenith 12.5% vs. Endurant 35.3%) even considering the limited follow-up of the Zenith group to two years as compared to five years for both ENGAGE and GREAT. All-cause mortality was similar in all three groups across the period examined while aorta-related mortality was uncommon. Reintervention rate was 15% at two years following the utilisation of the Zenith aortic graft while the rate of intervention at five years was broadly similar between ENGAGE and GREAT.

Conclusion

The newer generation, lower profile aortic endografts appear to have provided a safe and successful tool in the management of AAA in female patients, despite more complex aortic anatomy with shorter infrarenal neck length and larger aortic neck angulation.

## Introduction

Abdominal aortic aneurysm (AAA) is at least four times more common in males than females [1]. The natural history of aneurysm progression between the two sexes appears to vary significantly with inferior long-term outcomes in females following operative repair [2-6]. The underlying aetiology for this trend is unclear but several theories have been proposed.

Aortic aneurysms in females, of an equivalent dimension to their male counterparts, likely represent more advanced disease due to gender-based differences in mass [2]. They also appear to present later, with a larger size, and a preponderance towards more rapid progression as well as earlier rupture [7,8]. Furthermore, hormonal factors lead to a variation in atherosclerotic disease patterns between the two sexes [9]. This variation in natural history of aneurysm progression, combined with the under representation of females in studies of endovascular aortic aneurysm repair (EVAR), has led to a significant data deficit on optimal management of AAA in females. Guidelines from the Society for Vascular Surgeons (SVS) have acknowledged these differences with a suggestion of a treatment threshold of 5.0-5.4 cm in females as compared to greater than 5.4 cm in males [10].

EVAR is the preferred method for repair of AAA with a multitude of endografts commercially available. However, minimal long-term data has been published on newer generation devices. Three of the most prominent endoprostheses are the Endurant (Medtronic Vascular Inc, Dublin, Ireland), the Excluder (W. L. Gore & Associates, Flagstaff, AZ), and the Zenith (Cook Inc., Bloomington, IN). Each device has different accepted thresholds for specific morphologic features expressed in their instructions for use (IFU), particularly regarding the infrarenal neck length and angulation, although no gender specific recommendations have been outlined [11,12].

The altered aneurysm morphology present in female patients has been under represented in the data evaluated to formulate the current guidelines while the impact of endograft selection has not previously been assessed in this group. The aim of this study is to assess whether graft selection influences outcomes following AAA endoluminal repair in female patients.

## Materials and methods

Data source

Previously published data was obtained, and subsequently collated from three sources; the Endurant Stent Graft Natural Selection Global post-market registry (ENGAGE), the Global Registry for Endovascular Aortic Treatment (GREAT) and the U.S. Zenith multicenter trial in combination with the Zenith female registry [6,11,13].

ENGAGE (Clinicaltrials.gov identifier, NCT00870051) was designed to augment the knowledge base about endovascular aortic aneurysm repair in a real-world population implanted with the Medtronic stent graft system (Endurant, Medtronic, Dublin, Ireland) [13]. Patient enrollment in the ENGAGE registry occurred between 2009 and 2011, with a follow-up duration of 10 years. A total of 1263 patients from 79 international centres were enrolled with minimal inclusion criteria. Select exclusion criteria included probability of non-adherence to follow-up requirements, and concurrent participation in another trial that might confound results [13]. Data on outcomes following five years of follow-up was utilised within this analysis.

GREAT (Clinicaltrials.gov identifier, NCT01658787) was designed for the collection of data on the management of serious adverse events and follow-up patterns after the implantation of all Gore® aortic endografts (W.L. Gore Associates, Flagstaff, AZ) [6]. Patient enrollment in the GREAT registry occurred between August 2010 and October 2016. Over 5,000 patients from 114 international centres were enrolled, with broad inclusion criteria and minimal exclusion criteria reflecting the real-world use of devices, with a follow-up duration of 10 years [6]. Data on outcomes following five years of follow-up was utilised within this analysis.

Zenith data was abstracted from the U.S. multicenter pivotal study of the Zenith AAA endovascular graft (Cook Inc., Indianapolis, USA) and augmented with data from the Zenith female registry [11]. The design of both the female registry and multicentre study have been described previously [14]. Patient selection criteria for the registry was the same as for the standard risk group, except for gender, and patients were treated using the same methods with matching follow-up. A total of 366 patients were enrolled between 2000 and 2003, the minority of which were females [11]. Data on outcomes following two years of follow-up was utilised within this analysis.

Endoluminal prosthesis

The Endurant endograft, available since 2008, has a two-piece design with a nitinol M-shaped stent skeleton which is covered with polyester fabric. Proximal fixation is augmented by a suprarenal stent with anchoring pins. The Endurant II was introduced in December 2011 and differed from its predecessor regarding additional radiopaque markers and reduction in delivery system profile. A later design (the Endurant IIs) included a three-piece design, although this was only made available in 2014. It is not included in this analysis as inclusion in the ENGAGE registry ended in 2011 [12].

The Excluder Endoprosthesis is a modular-bifurcated system that has been available since 1997. The main body features eight nitinol anchors for infrarenal fixation while the endografts’ nitinol stent frame is covered by polytetrafluoroethylene (PTFE). In 2004, a structural change of the device was made with the addition of a low-permeability expanded PTFE sleeve to the graft composition, owing to high rates of sac growth with the original device. In 2010, the C3 Delivery System appeared, but no graft modifications were made [12].

The Zenith AAA endovascular graft is a modular system consisting of a bifurcated three-component device of self-expanding stainless-steel Z-stents sewn with monofilament polypropylene sutures to the covering woven polyester fabric and multiple ancillary components for individual length adjustment as well as occlusion devices. Proximal suprarenal fixation of the device utilizes a bare stent with barbs [11].

The Endurant is suitable for infra-renal necks ranging from 19-32 mm, allowing the treatment of infrarenal necks ≥ 10 mm in length if the infrarenal angulation is ≤60° and suprarenal angulation is ≤45°. It is also suitable for a neck length ≥15 mm with an infrarenal angulation of ≤75° and a suprarenal angulation of ≤60°. The Excluder is less liberal and is suitable for infra-renal necks ranging from 19-29 mm in diameter, ≥15 mm in length, with an infrarenal angulation of ≤60°. The Zenith is similar to the Endurant with suitability for infra-renal aortic necks between 18-32 mm, with an infrarenal neck length of ≥15 mm, and infrarenal angulation of ≤60° and suprarenal angulation of ≤45° [11,12].

Inclusion criteria

All female patients who underwent EVAR for AAA in the three aforementioned databases were included for analysis. The ENGAGE cohort included female patients who underwent intervention between 2009 and 2011, the GREAT recorded those whose intervention took place between August 2010 and October 2016, and the Zenith cohort included females undergoing intervention between 2000 and 2003. Outcomes were assessed according to all-cause mortality, aorta-related mortality (ARM), all reinterventions, any endoleak, Type Ia endoleak, conversions to open repair, and aneurysm rupture.

Statistical analysis

A retrospective analysis of prospectively recorded data from ENGAGE, GREAT, and Zenith was performed. Categorical variables are presented as frequencies with percentages. Continuous variables are presented as mean +/- standard deviation or as median and interquartile range (IQR). The x2 or Fisher exact tests were used for categorical variables, depending on sample size. P values < 0.05 were considered significant. All statistics were performed using SAS software (SAS Institute, Cary, NC).

## Results

Demographics and AAA characteristics

A total of 711 female patients underwent EVAR for AAA across the three studies. ENGAGE had 133 females (responsible for 10.5% of cases included in the registry), GREAT had 538 cases (14.3% of the total cohort), and Zenith had 40 females (10.9% of cases recorded within the database).

Baseline demographics for the female patients in all three registries are outlined in Table 1. The mean age was 75.7 (SD 7.1) years in ENGAGE, 74.7 (SD 9) years in GREAT, and 74.0 (SD 7.0) years in Zenith. Atherosclerotic disease was far more common in the GREAT cohort (coronary artery disease: ENGAGE 21.4% vs. GREAT 32% vs. Zenith 18% and peripheral vascular disease: 12.9% vs. 26.2% vs. 7.7%). Follow-up was performed as per the standard of care at each centre.

**Table 1:** Demographics of females who underwent EVAR for AAA in ENGAGE (Endurant Stent Graft Natural Selection Global post-market registry), GREAT (Global Registry for Endovascular Aortic Treatment), and the Zenith U.S. multicenter pivotal study. EVAR: Endovascular aortic aneurysm repair; AAA: Abdominal aortic aneurysm; n/a: data not available

	ENGAGE			GREAT			ZENITH		
Variable	Females N = 133	Males N = 1130	p-value	Females N = 538	Males N = 3220	p-value	Females N = 40	Males N = 326	p-value
Age	75.7 (7.1)	72.8 (8.1)	< .001	74.7 (9.0)	73.0 (8.3)	< .001	74.0 (7.0)	73.0 (8.0)	.28
Co-morbidity									
-Coronary artery disease	28 (21.4)	396 (36.4)	< .001	165 (32)	1327 (42)	< .001	7 (18)	120 (38)	.01
-Pulmonary disease	34 (26)	282 (25.4)	.887	176 (33.5)	747 (23.7)	< .001	14 (35)	74 (23)	.12
-Cardiac arrhythmia	20 (15.3)	179 (16.2)	.777	92 (17.5)	705 (22.3)	.013	4 (11)	74 (23)	.10
-Peripheral vascular disease	17 (12.9)	213 (19.1)	.080	137 (26.2)	571 (18.2)	< .001	3 (7.7)	57 (18)	.12
-Renal insufficiency	22 (16.5)	173 (15.5)	.745	100 (18.9)	483 (15.2)	.030	0	6 (1.9)	.99
-Coronary artery bypass graft	17 (12.9)	322 (28.9)	< .001	44 (8.3)	537 (16.8)	< .001	n/a	n/a	-
-Carotid disease	13 (11.6)	102 (10.8)	.800	69 (13.9)	373 (12.2)	.280	4 (10)	39 (12)	.99
-Stroke	4 (3)	63 (5.6)	.213	51 (9.6)	310 (9.8)	.911	n/a	n/a	-
-Congestive heart failure	9 (6.9)	62 (5.6)	.569	49 (9.4)	281 (8.9)	.727	1 (2.1)	29 (9.0)	.34
-Valvular heart disease	8 (6)	68 (6.2)	.946	42 (8)	241 (7.6)	.752	n/a	n/a	-
-Thromboembolic event	5 (3.8)	36 (3.3)	.734	28 (5.4)	197 (6.3)	.435	5 (13)	14 (4.3)	.04
-Transient ischaemic event	9 (6.8)	52 (4.7)	.276	39 (7.5)	172 (5.5)	.068	n/a	n/a	-

Anatomical variation at the time of intervention was minimal between all three groups with maximum aneurysm diameter greatest in those treated with the Endurant stent graft (ENGAGE 57.9 mm (SD 9.6) vs. GREAT 54.0 mm (SD 12.5) vs. Zenith 57 mm (SD 8.5)) (Table 2). Infrarenal neck angulation was greatest in the ENGAGE registry [38.1° (SD 26.2) vs. 36.8° (SD 25.1) vs. 31° (SD 19)]. Additionally, aortic neck length appeared to generally reside within the instructions for use (IFU) [24.5 mm (SD 11.9) vs. 27 mm (SD 15) vs. 29 mm (SD 13)]. The proportion of patients treated outside of instructions for use (IFU) was documented as 32.3% within the ENGAGE registry although this was not commented upon in the other two datasets.

**Table 2:** Anatomical characteristics of AAA. AAA: Abdominal aortic aneurysm; IFU: Instructions for use; n/a: data not available

Characteristics	ENGAGE			GREAT			ZENITH		
	Females N = 133	Males N = 1130	p-value	Females N = 538	Males N = 3220	p-value	Females N = 40	Males N = 326	p-value
Maximum AAA diameter (mm)	57.9 (9.6)	60.6 (11.8)	.012	54.0 (12.5)	55.8 (14.1)	< .001	57 (8.5)	57 (8.7)	.99
Aortic neck length (mm)	24.5 (11.9)	27.3 (12.4)	.014	27 (15)	29 (16)	.002	29 (13)	32 (16)	.49
Proximal neck diameter (mm)	21.8 (3.3)	23.9 (3.5)	< .001	22.8 (4.6)	23.9 (4.1)	< .001	23 (3.0)	25 (2.7)	.001
Distal neck diameter (mm)	22.9 (4.1)	25.1 (4.0)	< .001	22.1 (9.0)	24.2 (9.3)	< .001	n/a	n/a	-
Infrarenal neck angle (^o^)	38.1 (26.2)	29.4 (23.3)	< .001	36.8 (25.1)	29.7 (20.9)	-	31 (19)	21 (16)	.004
Right iliac artery diameter (mm)	12.9 (3.5)	14.3 (3.5)	< .001	10.1 (3.4)	12.1 (3.8)	< .001	11 (2.2)	14 (4.3)	< .001
Left iliac artery diameter (mm)	12.5 (2.9)	13.9 (3.5)	< .001	9.6 (2.9)	11.9 (3.6)	< .001	10 (2.2)	13 (3.3)	< .001
Outside of IFU (%)	43 (32.3)	182 (16.1)	< .001	n/a	n/a	-	n/a	n/a	-

Outcomes

Mortality, Rupture, and Conversion

The overall mortality was similar between GREAT and Zenith within the first two years of operative repair (10.6% vs. 12.5%) (Table 3). The five-year mortality between ENGAGE and GREAT was also found to be relatively similar (34% vs. 41.3%). Aneurysm-related mortality was low amongst all three groups, with two-year follow-up identifying no such cases within the Endurant cohort (ENGAGE 0% vs. GREAT 1.8% vs. Zenith 2.5%). Aneurysm rupture despite EVAR occurred in one documented case between the three studies (Zenith; secondary to type Ib endoleak) and was successfully treated via open repair. Requirement for conversion to open repair was not commented upon within the GREAT data but occurred within three cases (7.5%) in Zenith and one case (0.7%) in ENGAGE (Table 4).

**Table 3:** Mortality rates after endovascular aortic repair at different intervals. n/a: data not available

Time period	ENGAGE N = 133	GREAT N = 583	Zenith N = 40
All-cause mortality – n (%)			
<1 year	n/a	17 (3.4)	1 (2.5)
<2 years	n/a	48 (10.6)	5 (12.5)
<5 years	45 (34)	88 (41.3)	n/a
Aorta-related mortality – n (%)			
<1 year	0 (0)	8 (1.6)	0
<2 years	0 (0)	9 (1.8)	1 (2.5)
<3 years	0 (0)	9 (1.8)	n/a
<5 years	0 (0)	n/a	n/a

**Table 4:** Secondary outcomes including: Type 1A endoleak; Conversion to open aortic repair; Aneurysm rupture; and Surgical reintervention. n/a: data not available

Time period	ENGAGE, N = 133	GREAT, N = 538	Zenith, N = 40
Type IA Endoleak			
<2 years	n/a	n/a	0 (0)
<5 years	13 (10)	8 (1.5)	n/a
Conversion to open repair			
<2 years	n/a	n/a	3 (7.5)
<5 years	1 (0.7)	n/a	n/a
Aneurysm Rupture			
<1 year	0 (0)	n/a	1 (2.5)
<5 years	0 (0)	n/a	n/a
Surgical Reintervention			
<1 year	n/a	8 (1.5)	n/a
<2 years	n/a	20 (3.7)	6 (15)
<5 years	19 (14.3)	34 (6.3)	n/a

Endoleaks and Secondary Procedures

The five-year rates of any endoleak were significantly higher in the Endurant groups when compared to the Excluder (ENGAGE 35.3% vs. GREAT 6.7%) (Table 5). The two-year rate within the Zenith group was 12.5%. Similar timeframe data was not available for comparison with the other two cohorts. The majority of endoleaks within the GREAT study were type II (72.2%) although the precise number of such endoleaks was not disclosed in the other two studies. The rate of type 1a endoleak at five years was higher in the Endurant group as compared to the Excluder group (9.8% vs. 1.5%) although a considerable proportion of patients treated with the Endurant stent graft were treated outside of IFU (32.3%). Treatment within or outside of IFU was not recorded in GREAT or Zenith.

**Table 5:** Type of endoleak according to graft/registry. n/a: data not available

Variable	ENGAGE	GREAT	Zenith	ENGAGE	GREAT	Zenith
	Procedure			Total through 5 years		Total through 2 years
Subjects enrolled (n)	133	538	40	133	538	40
Endoleak – n (%)	28 (21)	1 (0.2)	11 (27.5)	47 (35.3)	36 (6.7)	5 (12.5)
Type Ia	2 (1.5)	0	n/a	13 (9.8)	8 (1.5)	0
Type Ib	n/a	0	n/a	n/a	8 (1.5)	1 (2.5)
Type II	21 (15.8)	1 (0.2)	n/a	n/a	26 (4.8)	n/a
Type III	1 (0.8)	0	n/a	n/a	0	0
Type I o Type III	3 (2.3)	0	n/a	20 (15)	16 (3.0)	1 (2.5)
Type IV	1 (0.8)	0	n/a	n/a	0	n/a

At the completion of two years of follow-up, 15% of patients had required surgical reintervention in the Zenith group as compared to 9% within the GREAT cohort. Two-year data from the ENGAGE registry was not available for comparison with the other two cohorts. Five-year outcomes highlighted an overall reintervention rate of 6.3% in the Excluder stent graft cohort as compared to 14% within the Endurant group.

## Discussion

The overall incidence of AAA has been shown to be up to six times greater in males than females [1]. The amalgamation of the three registries utilised in this analysis has identified that only 10-15% of cases were women [6,11,13]. Therefore, females are likely under-represented within the literature with limited data on long-term outcomes following the utilisation of the modern generation, lower profile, aortic stent grafts.

The newer generation endografts, with widely applicable IFU, have likely revolutionised the endovascular management of AAA in female patients. Previously, the smaller calibre access vessels as well as the more morphologically complex aortic anatomy have been identified as factors influencing outcomes following EVAR in this cohort [1,14]. However, the technical advances in endograft design which have resulted in increased conformability as well as wider IFU, has led to an improvement in both peri-operative and post-operative outcomes. This has been highlighted by previous analyses of the ENGAGE registry which identified similarly positive results between males and females treated with the Endurant stent graft despite more complex anatomy in the latter cohort [13,15]. This likely applies to the latest generation Cook and Gore aortic stents that are also included in this analysis, although the broader applicability of the Endurant in more complex aneurysms can be highlighted by the shorter median infrarenal aortic neck length as well as the greater median aortic neck angulation at the time of intervention in this analysis. This is also exhibited in the IFU as outlined in the methodology, with greater parameters for Endurant deployment [12].

Previous studies have highlighted a significantly increased rate of mortality in females after EVAR [16]. This was difficult to compare between these three registries given the variability in follow-up duration as well as the large proportion of patients lost to follow-up. The identification of an extremely low rate of aortic-related mortality, at both two and five years post stent implantation, appears to be extremely encouraging in regards to the safe and successful utilisation of these low-profile grafts. Amongst the analysed stents, there was only one (2.5%) aortic-related mortality in the Zenith cohort compared to nine (out of 538 cases, 1.7%) in the Excluder group. Interestingly, despite the greater proportion of complex aortic anatomy within the Endurant group, highlighted by 32.3% of patients being treated outside of IFU, not a single case of aortic-related mortality was identified in the five-year follow-up that was completed via the ENGAGE registry. Given that the purpose of aortic aneurysm repair is to prevent aortic aneurysm rupture, this was an extremely encouraging finding.

It has previously been well described that females have more hostile aortic neck anatomy with a greater propensity towards the development of a Type 1A endoleak [17,18]. In this analysis, this only occurred in a very small minority of patients treated with the Gore excluder (1.7%) while it was not recorded in the Zenith group. In comparison, female patients treated with the Endurant stent graft had a 10% rate of Type 1a endoleak development. It was difficult to ascertain whether or not this was due to endograft design or the patient population. The significant proportion of patients treated outside of IFU in ENGAGE (32.3%) was possibly a factor but equivalent data was not available for either GREAT or Zenith registries.

Despite the relatively high frequency of Type 1a endoleak in the ENGAGE registry, this did not appear to equate to a greater rate of aortic-related mortality. Additionally, the increased frequency of Type 1a endoleak in ENGAGE did not result in a significant disparity in the rates of surgical reintervention between Endurant and the Excluder stent graft, which were relatively comparable. Interestingly, two-year follow-up in Zenith highlighted that 15% of female patients underwent re-intervention as compared to just 3.7% in the GREAT registry at the same post-operative interval. The reasoning behind this disparity was likely multifactorial including the increasing exposure and associated development in technical expertise that occurred in the decade between these registries (Zenith was recorded between 2000 and 2003; GREAT was recorded between 2010 and 2016).

There are some limitations to this analysis. Firstly, it is a retrospective analysis of an amalgamation of previously published data. However, the data in question (ENGAGE, GREAT, Zenith) was collected prospectively on an international scale, which provides this analysis with the power of a large multicentre study representative of ‘real-world’ experience. Secondly, both ENGAGE and GREAT had a high number of patients lost to follow-up which is a limitation of both registries. Once again, this is likely representative of everyday experience with patient factors changing over time. Thirdly, the variability in endpoints recorded between the three registries somewhat limits the applicability of these findings. As highlighted, Zenith only completed two years as compared to five-year follow-up in GREAT and ENGAGE. The widely differing time periods across which each registry was formed likely results in an element of bias towards the more recent studies.

## Conclusions

The newer generation, lower profile aortic endografts appear to have provided a safe and successful tool in the management of AAA in female patients, despite more complex aortic anatomy with shorter infrarenal neck length and larger aortic neck angulation. This evaluation highlights the efficacy of the latest generation of endografts, but decisions regarding specific graft selection are likely beyond this current analysis, due to the limitations outlined. A further analysis, by means of a comparative randomised controlled trial between the three most prominent aortic stent graft manufacturers, would be extremely beneficial.
